# Trk Receptors and Neurotrophin Cross-Interactions: New Perspectives Toward Manipulating Therapeutic Side-Effects

**DOI:** 10.3389/fnmol.2017.00130

**Published:** 2017-05-03

**Authors:** Yazan Haddad, Vojtěch Adam, Zbyněk Heger

**Affiliations:** ^1^Department of Chemistry and Biochemistry, Mendel University in BrnoBrno, Czechia; ^2^Central European Institute of Technology, Brno University of TechnologyBrno, Czechia

**Keywords:** tropomyosin receptor kinase, neurotrophic tyrosine kinase receptor, neurotrophin, drug side-effect, molecular dynamics, molecular mechanics

## Abstract

Some therapeutic side-effects result from simultaneous activation of homolog receptors by the same ligand. Tropomyosin receptor kinases (TrkA, TrkB and TrkC) play a major role in the development and biology of neurons through neurotrophin signaling. The wide range of cross-interactions between Trk receptors and neurotrophins vary in selectivity, affinity and function. In this study, we discuss new perspectives to the manipulation of side-effects via a better understanding of the cross-interactions at the molecular level, derived by computational methods. Available crystal structures of Trk receptors and neurotrophins are a valuable resource for exploitation via molecular mechanics (MM) and dynamics (MD). The study of the energetics and dynamics of neurotrophins or neurotrophic peptides interacting with Trk receptors will provide insight to structural regions that may be candidates for drug targeting and signaling pathway selection.

## Introduction

From a clinical perspective, adverse therapeutic side-effects are symptomatic/phenotypic features that have a direct effect on patients by decreasing compliance and delay of recovery (Khawam et al., 2006; Kuhn et al., 2010). However, most side effects can be explained by the drug’s mechanism of action (Khawam et al., 2006; Widakowich et al., 2007) and thus, drug targets can be traced by their side-effect similarities (Campillos et al., 2008). Adverse side-effects are a common challenge for novel therapeutics. Developing strategies for side-effect manipulation requires an understanding of the target structure/function and signaling pathways.

Two decades ago, the enthusiastic prospects of developing clinical treatments based on neurotrophins were being hindered by the absence of knowledge on the signaling pathways activated by these ligands. Their side-effects (e.g., pain, particularly at the injection site) were noted early in clinical trials on neurodegenerative diseases (Thoenen and Sendtner, 2002). Tropomyosin receptor kinases, also known as neurotrophic tyrosine kinase receptors (Trk), also modulated by the p75NTR receptor, play an essential role in the biology of neurons by mediating neurotrophin-activated signaling. Neurotrophins include nerve growth factor (NGF), neurotrophin-3 (NT-3), neurotrophin-4 (NT-4), brain-derived neurotrophic factor (BDNF), neurotrophin-4/5 (NT-4/5), neurotrophin-6 and neurotrophin-7 (Ultsch et al., 1999). Three Trk receptors (TrkA, TrkB and TrkC) are localized on the plasma membrane of the axon end, where they are activated and transported in vesicles to the body of neurons. Activated Trk receptors trigger signaling pathways in both the axon end and main neuron body. Several protein complexes play a role in the transport of Trk receptors to the axon (Arimura et al., 2009) and from the axon to the neuron main body (Heerssen et al., 2004). Trk receptors suppress apoptotic pathways through at least two mechanisms (Ras/PI3K/Akt and MEK/MAPK pathways). On the other hand, p75NTR directly induces apoptosis via the JNK-p53-Bax pathway and can modulate Trk activity by activation of NF-κB (Kaplan and Miller, 2000). Cross-talks between Trks and p75NTR are important for the development, maintenance and repair of the nervous system.

**Figure 1:**
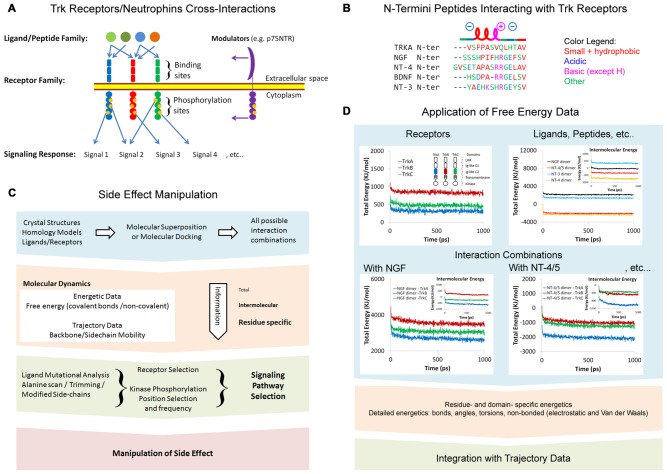
** (A)** Tropomyosin receptor kinase (Trk) receptors/neurotrophins cross interactions. **(B)** N-termini peptides interacting with Trk receptors showing similar α-helical and charge properties. Multiple alignments were modified from Clustal Omega alignments (EMBL-EBI web tools). **(C)** Side-effect manipulation strategy. **(D)** Application of free energy data. Representative energetics of the Trk receptors Ig-like C2 domains and neurotrophins were conducted via molecular dynamics (MD). Data shown are for demonstration purposes only. Structural energy was minimized by 5000 steepest descent steps and dynamics were performed at 2.5 femtosecond (fs) time intervals at 25°C to a total time of 1 nanosecond (ns) in Ascalaph Designer v.1.8.94. PDB ID for structures used here are: 1WWA, 1WWB, 1WWC, 1B98, 2IFG, 3BUK and 1HCF.

The simultaneous activation of several Trk receptors, of the three classes (TrkA, TrkB and TrkC) at the same time, is a result of a wide range of cross-interactions between Trk receptors and neurotrophins (Figure 1A). Trk-neurotrophin interactions depend on many factors such as binding affinity, abundance, selectivity and extracellular/intracellular modulation by other proteins (e.g., p75NTR). Recently, a number of peptides derived from the N-terminus tails of neurotrophins have been shown to mimic their parent molecules (Figure 1B). We will discuss this matter in a separate section. Here, we discuss new perspectives on the manipulation of side-effects via a better understanding of cross-interactions at the molecular level, using computational methods (Figure 1C). Available crystal structures of Trk receptors and neurotrophins are a valuable resource for molecular modeling studies. Computational studies are the rational follow-up for structural analysis, as well as prior to functional analysis. This intermediate role makes it an attractive strategy for targeted therapy and drug design. The approach presented here is also applicable to a wide range of receptor/ligand families. Trk-neurotrophin interactions lead to symmetrical receptor dimerization, followed by different conformational changes in the Trk intracellular kinase domain. The resulting kinase trans-phosphorylation process can vary in frequency and residue positions, and hence, vary in response and function. A phosphorylation map of at least four classified general cellular/phenotypic responses of TrkA has highlighted Y490 and Y785 as the most significant phosphorylation sites (Bradshaw et al., 2013).

Computational methods that exploit all structural combinations of receptors and ligands can be used to collect valuable data regarding the free energy and mobility of particular residues in the receptors and ligands. Mutational and functional analysis (either *in silico* or *in vitro*) must follow to identify and manipulate specific residues that control signaling pathway selection.

## Molecular Dynamics: Energetics and Mobility

The computational study of the free energy of molecular structures is done using molecular mechanics (MM). Force field functions/parameters are considered axiom for calculation of the potential energy of atoms and understanding the changes in covalent and non-covalent (non-bonded) interactions. Bonded energy results from covalent bonds (bond lengths, angles, torsions and improper chirality). Non-bonded energy results from electrostatic (ionic and hydrogen bonds) and Van der Waals forces. Theoretical understanding of the behavior of molecules and proteins in physiological environments within a simulated period of time can be computed through molecular dynamics (MD). Atomistic MD employs the free energies of atoms—calculated from the force field—and predicts their trajectories in femtosecond (fs) intervals, simultaneously and many times. MD is often performed in three steps: “minimization” of energy adapts the structure to the force field, then the “equilibration” step adapts the system to the temperature of interest and finally, a “production” step is used for MD computations. MD gives insight on several aspects of molecular phenomena, and has played a key role in our understanding of conformational changes in proteins, catalysis and binding sites in enzymes, as well as the allosteric effect in protein complexes. One particular aspect that is addressed in this article is an understanding of comparative protein-protein interactions and how it can be used to manipulate unnecessary interactions such as those resulting in side- effects. The main output of MD simulations includes information on the energetics of the system, as well as the trajectories of each atom over a specific period of time. Trajectories (the position and velocity vectors of atoms) can be used to identify particular regions of protein receptors that play a role in selectivity. Receptor-ligand comparative analysis of similar receptors of the same family (such as the Trk receptors) can provide insight to specific regions that exhibit divergent functions. Without MD analysis, these divergent regions are not always predictable by sequence and structure alone, yet are excellent candidates for the development of selective therapeutics.

The extracellular portion of the Trk receptors consists of the Ig-like C2 domain, the Ig-like C1 domain and the Leucine Rich Repeat domain (LRR). X-ray crystallography studies highlight the role of the Ig-like C2 domain in TrkA-NGF interactions (Wehrman et al., 2007) and TrkB-NT-4/5 interactions (Banfield et al., 2001). All possible interaction combinations between Trk receptors and neurotrophins can be computationally studied via molecular superposition and molecular docking (Figure 1C). The extracellular domains that lack experimental crystal structures, including those of TrkB and TrkC, can be constructed via homology modeling (Haddad et al., 2017). Homology modeling is a well-established method that can be used to construct structures for unknown domains or proteins, based on known similar and homolog structures. It is important to note that some of the Ig-like C2 structure binding neurotrophins are missing in the model PDB ID: 1WWA (Ultsch et al., 1999). However, it is well represented in later crystal structures, i.e., PDB ID: 2IFG (Wehrman et al., 2007).

The Trk binding sites on some neurotrophins are predicted from their known structures, such as NT-3 (Gong et al., 2008), NT-4 and NT-4/BDNF heterodimer (Robinson et al., 1999). Understanding the selectivity and binding energetics of Trk receptors with these neurotrophins can be achieved through protein-protein molecular docking, followed by MD. There is evidence of co-expression of many neurotrophins, as well as evidence that most of them can form heterodimers, at least *in vitro* (Robinson et al., 1999). If such phenomena are common in physiological conditions, then the role of heterodimers in the activation of Trk receptors will need revisiting. Due to the known structures of many neurotrophins, this issue is also attractive to computational studies.

In order to simulate each Trk-neurotrophin combination, large computational resources are required. Each MD simulation can involve calculations of trajectories for more than 100,000 atoms in fs intervals for hundreds of nanosecond (ns). Solvation energy, arising from hydrogen bonds of water molecules, plays a significant role in protein-protein interactions. An alternative computational approach (implicit solvent model) allows for calculation of solvent effects without using explicit water molecules. Hence, it provides a trade-off between used computational resources and accuracy. Coarse-grained MD is another approach that provides a trade-off by simplifying atomistic molecules to lower-resolution models at various “granularity” levels.

## Energetics of Trk Receptors and Neurotrophins

A comprehensive approach is required for studying the energetics of all interaction combinations between the Trk receptors domains and neurotrophins (Figure 1D). Free energy is an indicator of the spontaneity of interaction. All free energy calculations are based on the force field function and parameters, which themselves are based on experimental knowledge (Ponder and Case, 2003). The intermolecular energy between two or more chains of proteins is a direct representation of binding energy. Negative free energy indicates high stability, while positive free energy indicates instability. However, the energy values presented in the flowchart in Figure 1D were rough estimates calculated in a vacuum space and using few computers for several weeks to produce simulations on a picosecond (ps) scale. To set up a proper MD simulation, it is important to consider numerous factors representing physiological conditions. These include a water solvent model, ions, the protonation state of amino acids, and glycosylation. The changes in all energy components, including covalent and non-bonded energy, can be profiled for all residues in the protein. This detailed profile can later be correlated with trajectories to provide a comprehensive understanding of the protein regions involved in interaction.

## Atomic Trajectories and Selectivity

**Figure 2:**
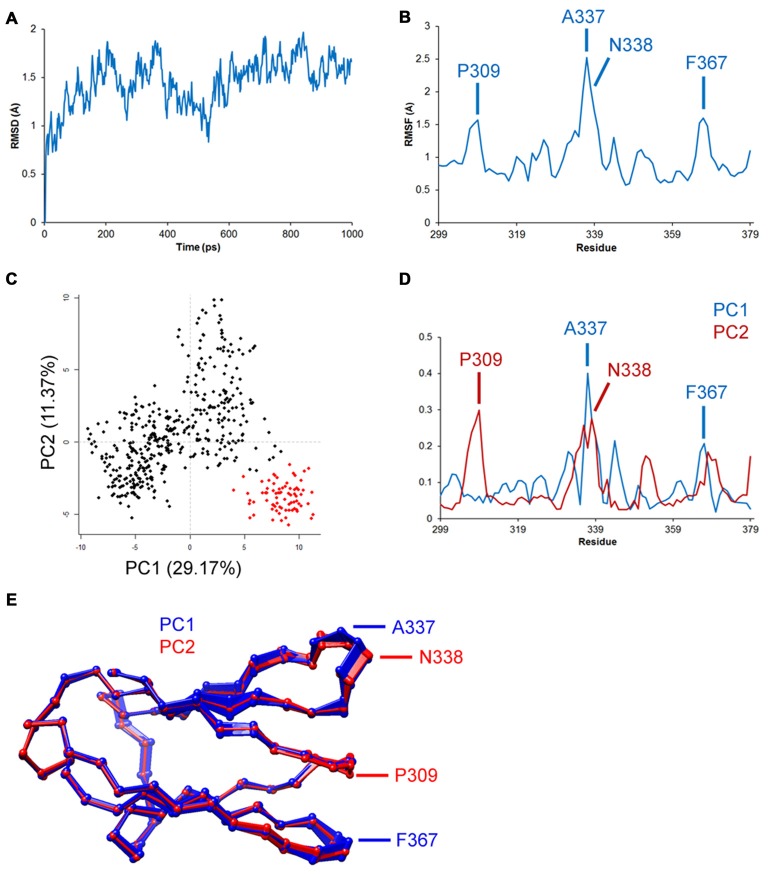
**Representative trajectory analysis of the TrkA receptor Ig-like C2 domain’s (PDB ID: 1WWA) simulated dynamics in Amber v.14.** Data shown are for demonstration purposes only. Structure was prepared using CHARMM-GUI server and placed in a TIP3 water model and periodic boundary box with 0.15 M KCl. Structure was minimized and equilibrated, and simulations were conducted in 2 fs time intervals at 37°C for a total of 1 ns. Trajectory analysis was done using R-language Bio3D tools. **(A)** Root-mean-square deviation (RMSD) analysis shows deviation of 1–2Å from the original structure. **(B)** Root-mean-square fluctuation (RMSF) analysis shows the highest fluctuations in residues P309, A337, N338 and F367. **(C–E)** Principal component analysis shows two distinct clusters of trajectories, which may indicate two alternative foldings, particularly in the aforementioned residues.

As described previously, atomic trajectories are the raw output of MD and provide valuable insight to the mobility of specific regions in a protein during simulation. Comparative study using the known atomic coordinates of two structures or the same structure in two time intervals can be done using the root-mean-square method. Root-mean-square deviation (RMSD) of distance between corresponding atoms of two structures provides a quantitative estimate of structural differences/changes (Tsai, 2003). An example showing some of the advantages of trajectory analysis is featured in Figure 2. RMSD of the carbon-alpha atom in Figure 2A represents the average deviation in backbone folding of the Ig-like C2 domain of TrkA from its original structure. The deviation of backbone increased to ~2Å before retracting to 1Å and then fluctuated between these two numbers. MD simulations are often extended to relatively long periods (e.g., >200 ns) until a steady RMSD is reached in order to draw reproducible findings and reliable conclusions. Root-mean-square fluctuation (RMSF) describes the average changes per amino acid during the entire period (Figure 2B). In the example, RMSF peaked at several regions, particularly around the residues P309, A337, N338 and F367. These peaks indicate high mobility of the backbone at these regions, which are on the interface with the neighboring Ig-like C1 domain. Principal component analysis of the trajectories can be used to describe alternative conformations of structure that are correlated together as separate components/dimensions (Figures 2C–E). Clustering principal component analysis indicates two distinct samplings of trajectories in the example (Figure 2C). The contribution of each residue to the first two components is shown in Figure 2D. As expected, the residues with previous high RMSF showed distinctly correlated trajectories, which may represent alternative folding (Figure 2E). The investigation of atomic trajectories after simulation of Trk receptor-neurotrophin interacting together in physiological conditions will provide insight to regions responsible for selectivity. Current technical challenges—in addition to computational resources—include quality of structure, the protonation state of amino acids and glycosylation of asparagine residues. An alternative computational method for the study of functional mobility in large structures is normal mode analysis (NMA). NMA depends on calculation of the independent harmonic motion of molecules and does not require trajectories. Here, the regions of lowest frequencies, *viz.*, soft modes, show the most movement and are most relevant to protein functions (Skjaerven et al., 2009).

## Role of p75NTR

The p75NTR receptor is foreseen as a major contributor to Trk signaling and must therefore be included in the discussion. This receptor interacts strongly with uncleaved neurotrophins (pro-neurotrophins) and with their activated forms, but with lesser affinity. The ways have been described in which p75NTR can alter Trk functions: (1) altering binding specificity; (2) altering binding affinity; and (3) altering Trk kinase activity directly in the cytoplasm (Greene and Kaplan, 1995). The published crystal structure of p75NTR binding NT-3 shows a symmetrical complex with accessible Trk binding sites on NT-3 (Gong et al., 2008). In fact, structural modeling showed that p75NTR can fit well in a ternary complex of p75/NGF/TrkA (Wehrman et al., 2007). Unfortunately, such a ternary complex has to date failed to be confirmed experimentally (Barker, 2007). Additionally, interaction of p75NTR with the Sortilin receptor has been indicated to play a role in its affinity with pro-neurotrophins via the extracellular domains of these receptors (Skeldal et al., 2012). Both p75NTR and Sortilin receptors play a role in pro-neurotrophin-induced apoptosis in neuronal cells (Jansen et al., 2007). A proNGF/sortilin/p75NTR ternary complex has also been reported (Feng et al., 2010). Researchers support the hypothesis that neurotrophins bind lesser affinity receptors prior to being introduced to Trk receptors. Lesser affinity receptors, similar to p75NTR, can explain the strange kinetic behavior of p75NTR/NGF and should be identified and knocked out in experiment settings. The aforementioned computational methods can also be used to shed light on this cross-interaction network.

## Neurotrophic Peptides

Several molecules that can mimic the signaling of neurotrophins have already been confirmed in clinical trials (Price et al., 2007). Mimicking neurotrophic peptides derived from the N-termini of neurotrophins show significant therapeutic potential. The peptides share similar properties including a short α-helix turn, prompted by a proline residue or alternating acidic/basic residues (Figure 1B). Experimental and computational evidence indicate the role of N-termini of NGF and NT4 in the recognition of neurotrophins by Trk receptors (Stanzione et al., 2010). Neurotrophic peptides based on the first 14 and 12 N-terminal residues of NGF and BDNF, respectively, were recently reported (Travaglia et al., 2015; Satriano et al., 2016). These peptides trigger some but not all the signaling pathways activated by their parent neurotrophins, and have interesting affinities with Zn^2+^ and Cu^2+^ (Travaglia et al., 2011, 2015). Interestingly, crystallization of TrkA Ig-like C2 domains formed dimers with their N-termini, and were binding at the same neurotrophin binding site (Ultsch et al., 1999). The fact that this sequence aligns well with neurotrophic peptides is intriguing (Figure 1B). It is believed that these peptides may activate TrkA receptors in a p75NTR-independent fashion. There are two ways in which to explain Trk receptor trans-activation by peptides: first, the binding of peptides can induce or reduce the mobility of certain regions of the receptor, resulting in conformational changes in receptor and stabilization of helical secondary structure of peptide. Second, it is more likely that peptides will cluster together and enable the dimerization and trans-phophorylation of Trk receptors. Further evidence is required to exploit Trk receptor specificity, as well as the therapeutic benefits of these peptides and other N-termini pertaining to other neurotrophins. Short peptides are extremely attractive for computational studies and can have significant therapeutic potential. Full peptide characterization with trimming and alanine-scan is required to optimize their therapeutic abilities. We hope that investigation of the N-termini of other neurotrophins and the signaling pathways they trigger will shed more light on their potential benefits in targeted therapy.

## Future Prospects

Neurotrophins are highly prospective therapeutics for many types of neurodegenerative diseases and cancers. Therapeutic side-effects remain a challenging aspect of developing treatment. Manipulation of therapeutic side-effects is possible when signaling pathways can be selected at the molecular level. Comparative MD studies that identify residue-specific changes in energetics and mobility can highlight structural regions with selective functional roles. Beyond dynamics and energetic profiling, the future prospects of side-effects manipulation lie in direct functional analysis. Directed mutational analysis (*in vitro* and *in silico*) will shed light on binding mechanisms controlling kinase activity and phosphorylation. It is also important to take into consideration the various factors affecting Trk receptors and neurotrophin cross-interactions. These include quantitative expression and co-expression of Trk receptors, alternative splicing of Trk receptors, co-expression with modulators (e.g., p75NTR receptor), co-expression of neurotrophins and the frequency of heterodimers, and factors affecting the transport of Trk receptors to and from axonal plasma membrane.

The case of N-termini peptides serves as a good example of selective pathway activation and requires less computational resources for study. This field remains full of surprises. An alternative hypothesis suggests the activation of Trks by peptides to be dimerization independent (i.e., modulator dependent). However, it is important to remember that neurotrophins’ N-termini are not the only part interacting with Trk receptors. On the other hand, the LRR and Ig-like C1 domains may also be involved in neurotrophin interaction. Computational methods can be utilized with the available structures to shed new light on this network.

It is obvious that *in vitro* experiments in the field of neurotrophin signaling are becoming more difficult to control. Good expression profiling of neurotrophins, Trk receptors and modulators is more laborious yet necessary for well-controlled experiments. We hope that progress in computational-based studies on neurotrophin signaling will help to illuminate the mysteries of this network.

## Author Contributions

All authors contributed to the design of work. YH wrote the manuscript. VA reviewed the manuscript and ZH was principle investigator and contributor to the scheme and organization of the work.

## Funding

We gratefully acknowledge the Czech Agency for Healthcare Research, AZV (15-28334A) and the Ministry of Education, Youth and Sports of the Czech Republic under the project CEITEC 2020 (LQ1601) for financial support of this work. Computational resources were provided by the CESNET LM2015042 and the CERIT Scientific Cloud LM2015085, provided under the program “Projects of Large Research, Development and Innovations Infrastructures”.

## Conflict of Interest Statement

The authors declare that the research was conducted in the absence of any commercial or financial relationships that could be construed as a potential conflict of interest.
